# A Design for Safety (DFS) Framework for Automated Inspection Risks in Metro Stations by Integrating a Knowledge Base and Building Information Modeling

**DOI:** 10.3390/ijerph20064765

**Published:** 2023-03-08

**Authors:** Ping Liu, Yongtao Shang, Lei Zhang

**Affiliations:** 1 School of Civil Engineering, Lanzhou University of Technology, Lanzhou 730050, China; 2 Smart City Research Center, Nanjing Tech University, Nanjing 210096, China

**Keywords:** metro stations, design for safety, knowledge base, risk inspection plug-in, building information modeling

## Abstract

Safety issues have always been of great concern to the metro construction industry. Numerous studies have shown that safety issues are closely related to the design phase. Many safety problems can be solved or improved by developing the design. This study proposes a structured identification method for safety risks based on the metro design specifications, journal literature, and expert experience. A safety knowledge base (KB) for the design was established to realize safety knowledge sharing and reusing. The KB has been developed into Building Information Modeling (BIM) software as an inspection plug-in to achieve automated analysis and retrieval of safety risks. The designers are provided with a visualization of risk components to locate and improve the pre-control measures of the design. Subsequently, the process of design for safety (DFS) database creation was demonstrated with a metro station project, and the feasibility of applying the KB to safety checking in BIM was verified. In response to the inspection results, safety risks in the construction phases can be eliminated or avoided by standardizing and improving the design.

## 1. Introduction

With the growth of population and the expansion of city scale, the metro has become an important way to solve transportation problems. The metro construction industry has been well developed in China. According to the China Urban Rail Transit Association, the number of metro lines in operation has increased by 229, and the mileage in operation has reached 7209.7 km. Currently, the scale of planned and under-construction lines is still growing [1]. However, the complexity of the construction environment of metro stations leads to frequent occurrences of various safety accidents. According to incomplete statistics by Yu [2], 246 metro construction safety accidents occurred in China from 2002 to 2018, with 280 fatalities. The idea that the design phase has the potential to improve life-cycle safety performance is widely accepted [3,4,5]. Studies have shown that many safety accidents can be avoided by improving the design, effectively improving safety management quality [6,7,8]. Xiahou et al. [9] collected 442 accident cases from the whole life-cycle of metro and found that 236 accidents were related to safety design. By studying 224 engineering incidents with casualties, Gambatese et al. found that 42% were associated with design for safety (DFS) and that risk events could be avoided through DFS [3,10]. Therefore, DFS is an effective way to eliminate hazards before metro construction [11].

The philosophy of considering security in the design phase is consistent with the DFS. The concept of DFS first published by the National Safety Council in 1995. The DFS refers to eliminating or avoiding risk by specifying and improving design before the risk occurs [12,13]. Its core idea is the preventive safety hazard in the design phase [14]. The DFS, as a “preventive” and “proactive” design concept, provides a theoretical basis for reducing metro safety incidents during the design phase [15]. Information technology plays an essential advantage in DFS, such as safety knowledge (SK) and Building Information Modeling (BIM). Knowledge base (KB) can address the inadequate knowledge of safety managers. In addition, KB can improve the sharing and reuse of knowledge and experience in engineering practice and reduce the loss of engineering SK. BIM technology provides parametric and visual models and has great potential for information exchange and compliance checking [16]. Some specific types of safety incidents can already be solved using BIM in the design phase, such as falling from height. Previous studies have shown that it is feasible to use BIM and KB technologies to implement DFS in the metro design phase.

This study establishes a KB for the design process using the SK of metro stations. In addition, the KB is integrated with BIM to develop a plug-in for automatic inspection, which enables safety hazards to be identified and eliminated in the design phase. The remaining parts are described below. Section 2 is an introduction to the existing research on BIM and KB. Section 3 introduces the framework of the safety KB and the automated inspection plug-in. Section 4 is used to construct the DFS-KB for metro safety design. Section 5 combines the KB with the BIM platform to achieve an automatic safety inspection (SI) of design results and visual output of risks. Section 6 introduces the KB building process using a metro station case. Moreover, one of the rules is used to verify the effect of applying the KB to the BIM model for safety checking.

## 2. Literature Review

### 2.1. Knowledge Management for DFS

The knowledge management process usually includes acquisition, storage, representation, and transfer [16]. Through these processes, structured knowledge can be stored in the KB for easy retrieval by designers and safety managers, and the knowledge reuse can be improved. KB is one of the main tools used for knowledge management. In general, the primary role of a KB lies in the representation, storage, transfer, reasoning, and acquisition of knowledge.

The emergence of information technology has made the sharing and reusing of SK possible. With the application of information technology in the construction field, some scholars have focused their research on transforming safety specifications into a form that can be recognized, stored, and executed by computers [17,18]. Zhang and El-Gohary [19] proposed a natural language processing approach, based on a combination of semantics and machine learning, to extract SK automatically from the construction specification text. Malsane et al. [20] analyzed residential fire safety-related specifications, in order to translate human-readable textual rules into computer-executable rules. Guo and Goh [21] used ontology language to structure fall accident SK, in order to facilitate knowledge sharing and reuse. In addition, the KB was gradually applied to security design. In 1999, Gambatese and Hinze [22] created a safety design toolbox called “Design for Construction Safety Toolbox”, which was the first design aid based on a database of safety recommendations. For fall accidents in the construction industry, Guo and Goh [23] created a KB of 50 stored cases, which help designers address fall safety issues. Moreover, safety KB has shown unique advantages in visualizing and dynamizing metro construction safety. Zhang et al. [24] proposed a safety ontology-based method for organizing, storing, and reusing construction SK to visualize job risk analysis. Dong et al. [25] developed a dynamic knowledge flow atlas for SK management in metro projects. Therefore, knowledge-based decisions are helpful in helping engineers improve the quality of their designs.

Despite the advances in information technology and knowledge management in the construction industry, there is still a fragmentation between SK management and design work [24]. Presently, designers use their SK to identify inspection risks from CAD (two-dimensional) drawings [26]. Security risk analysis mainly relies on the inspector’s experience and understanding of the specifications. However, this approach requires a large number of staff, material, and time, and is highly subjective, making the accuracy of risk identification difficult to control [27]. A better approach is to assist engineers in automating hazard detection tools, shifting the focus of engineers’ attention to problem-solving rather than problem-detection [28].

### 2.2. BIM Tool for DFS

In recent years, the visualization, sharing, and extensibility of BIM technology have rapidly made it become an important management platform for the metro industry [29]. BIM technology enables the automatic identification of risks in the 3D model and the visualization of risk output, features that KB does not have. In addition, there are insufficient people to perform SI during the design phase. Traditionally, the DFS measures required in design specifications may be neglected by designers and safety managers, which results in safety defects including unreasonable design solutions, unsuitable safety protection facilities and equipment, and non-compliance with the specifications [12].

BIM is widely used in engineering construction management [30,31], engineering information management [32,33], and engineering safety management [26,34]. Especially in engineering safety management, BIM technology has shown unique advantages in DFS [35,36]. For example, the use of BIM technology for automatic safety compliance reviewing [20,37], combining BIM technology with database technology, etc. [11]. The use of information technology has changed the traditional way of safety management in the metro construction industry. It provides a new idea for improving design safety. However, understanding buildings’ spatial location relationships is a challenge for engineers during safety risk inspections. Decision makers must have a solid 3D spatial understanding to master complex spatial transformations [28]. The new generation of BIM technology engineers lacks education and training. Therefore, some scholars have focused their research on developing SI systems in the design phase. Qi et al. [38] designed a construction SI tool to explore how to check worker safety issues in design models based on existing rules. Chantawit et al. [39] combined safety management and virtual environments to help safety managers analyze and utilize safety planning. There are also scholars who study how to combine BIM and KB technology to solve engineering safety problems. Zhou et al. [5] explored the relationship between construction safety and information technology. They explored the use of databases, building information models, and sensing technologies in construction safety management. Hossain et al. [12] combined BIM with a structured DFS database to improve designers’ ability to process risk information. Yuan et al. [16] developed a Prevention Through Design plug-in for the KB to implement automated inspection functionality.

### 2.3. Research Gap

In prior studies, several DFS-KBs are developed to store accident cases, organize building safety specifications, and assist managers in decision-making. However, most of them focus on buildings, with little attention paid to metro stations. Meanwhile, the inspection process based on DSF knowledge is tedious and inefficient, which can not eliminate quality issues in metro station construction. Integrating KB and BIM with DFS in metro station can support engineers’ and safety managers’ decision-making in complex and uncertain situations. Therefore, it is of great theoretical and practical significance to integrate KB and BIM, in order to solve the safety problems of metro stations.

## 3. Framework

### 3.1. Research Framework

**Figure 1:**
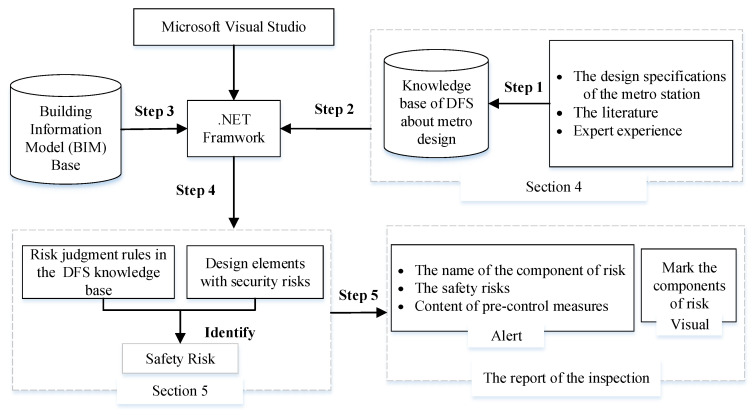
Framework of this research.

This study aims to realize the structured storage of DFS knowledge, build a DFS-KB for metro design, and apply the KB to the BIM automated SI process. The research mainly consists of two parts: The first is to establish a DFS-KB based on the metro design specifications, journal literature, and expert experience; the second uses the safety KB to develop an automated 3D model SI plug-in integrated with Revit. These two parts are presented in Section 4 and Section 5 of this paper. The basic framework of this study is shown in Figure 1, which consists of five steps.

Step 1: Identify the DFS knowledge in the metro design specifications, journal literature, and expert experience, which are used to build the DFS-KB. It mainly includes three parts: (1) Design safety risk identification; (2) Extraction of pre-control measures; and (3) Pre-control measure and risk linkage.

Step 2: Query the rule information for SI from the KB.

Step 3: Query the component design information for safety checking from the BIM information base.

Step 4: Develop the rule inspection plug-in. The safety hazard can be inferred by confirming whether the design follows the rules in DFS-KB.

Step 5: Output the inspection result. The result includes the risk alerting window and the risk visualizing marker.

In step 4, the components that violate the DFS knowledge are identified. In step 5, the components in the design model that violate the design requirements are displayed, and the treatment options that can improve the design are queried from the DFS-KB. The designers and safety managers can improve the design based on the measures provided and eliminate the risks in the design phases.

### 3.2. DFS-KB

Microsoft SQL Server is often used to create, organize, manage, and query databases. It has an open interface that can be linked to the BIM model database, and access to the database data is achieved through Structured Query Language (SQL) statements. Therefore, this paper selected Microsoft SQL Server 2008, a relational database, to build the KB. SQL is a database query and programming language for accessing data. In addition, it can be used to query, update, and manage database systems. In this study, SQL is used as the programming language for database creation, querying, and maintenance.

**Figure 2:**
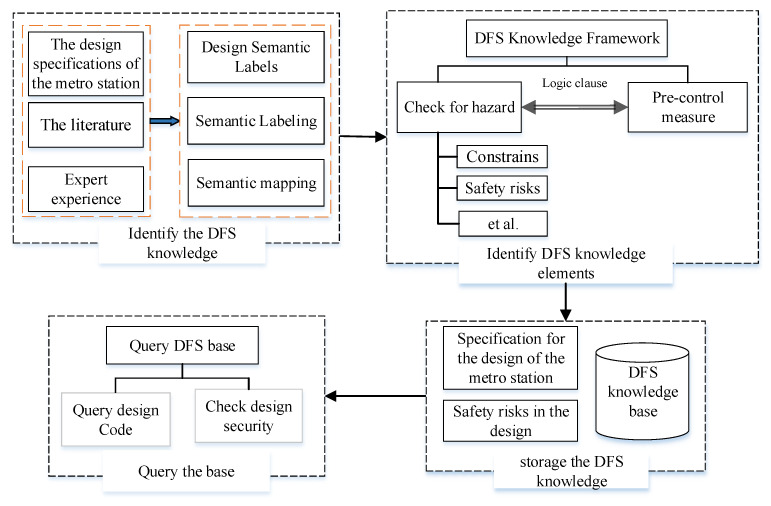
Creation process of the DFS-KB system.

The established KB can realize the information query of DFS knowledge in design. In addition, it lays the data foundation for realizing the automated SI of BIM models. The metro design specifications, journal literature, and expert experience are the condensation of valuable experience from previous engineering practice, and their accuracy has been proven in the application of engineering practice. This study establishes a KB for the SK among them, which provides an informative way to retrieve DFS knowledge. The KB consists of three main parts: (1) The specification for design of metro; (2) Design safety risks; and (3) Pre-control measures. The KB establishment process is shown in Figure 2, which mainly contains the following four steps.

(1) Identify the DFS knowledge. A wealth of SK is provided in the metro design specifications, journal literature, and expert experience. Since design specifications are in different forms, the first step is to simplify these rules and organize them in the same format. The SK describes the constraints of design elements under different design conditions.
(2) Identify DFS knowledge elements. Most of the DFS knowledge exists as a type of attribute constraint. In this process, it is necessary to determine the six DFS knowledge elements in the constraint class specification: Design Topic, ELE, PRE, Constraints, Safety Risks, and Pre-control Measures. In addition, the identified DFS knowledge elements are connected in the form of logical sentences.
(3) Building DFS-KB. The KB contains two types of tables to store the DFS knowledge: The specification for the metro station table design and the metro station’s safety risk table. The combination of knowledge between the tables is achieved by establishing inter-table relationships. Then, the data in the specification for the metro station table design can be accessed through the metro station’s safety risk table.
(4) The query of DFS knowledge. Access to DFS knowledge in the KB can be achieved by writing a “SELECT” statement. The results can be displayed in the KB directly or in Revit’s knowledge interface.

### 3.3. BIM-Based Automatic Inspection Tool

BIM technology is often used in the design phase. Safety hazards can be visually demonstrated when safety issues are combined with BIM models [40]. Therefore, in this paper, the DFS-KB is integrated into BIM software to achieve the automated SI of BIM models. There are two main ways to automate SI: One is to use the IFC-based model checker (e.g., Solibri Model Checker or BIM Server) [16]. The other is to develop plug-ins that can be used in BIM software. The second approach is used in this paper. The developed program runs using the External Command provided by Revit. There is no need to rely on third-party software, which makes the retrieval process more convenient. Eventually, the visualization of risk components can be located, and the output of pre-control measures can be realized.

Revit, developed by Autodesk, shows unique advantages in BIM applications. Its External Command “Add-In Manager” provides excellent scalability. Therefore, in this paper, Revit is used as the application platform for automatic inspection plug-ins. The NET Framework provides the development environment required for inspection tools, allowing for seamless integration between different development languages. C# is a commonly used desktop application development language with good practicality. Therefore, this paper uses C# to complete the development of the automated inspection plug-in.

The developed plug-in can be run through the External Command “Add-In-Manager” provided by Revit. The result of the check is presented in two forms: (1) The risk alerting window. The contents displayed in the alarm window include the names of risk components, safety risks, and pre-control measures. (2) The risk visualizing marker. The components with design risks are displayed in the BIM model in highlighted colors.

## 4. Establishment of a DFS-KB

Knowledge of DFS in natural language form is usually distributed in the metro design specifications and journal literature [15]. Nevertheless, the SK accumulated by engineers on the job site cannot be neglected [16]. Structured design and representation in national standards and journal literature can address some security concerns [26]. Explicit knowledge is usually obtained directly from manuals or literature. The knowledge and experience accumulated by experienced experts or engineers in engineering practice are generally referred to as tacit knowledge. This knowledge can be used as a supplement to explicit knowledge [28]. As the design rules are in different forms, they need to be first reorganized into the same format.

### 4.1. Identifying DFS Knowledge

The DFS specifications contain both structured and unstructured knowledge. Building a KB requires structured knowledge. Therefore, structured knowledge is selected in this paper. The specifications are preprocessed before the structured design, and the text in the design specification is cut into individual sentences according to semicolons, periods or semantics [41]. Moreover, it ensures that the segmented sentences can express the complete semantic specification. The DFS specification mainly contains two types of rules [18]: (1) Quantitative requirement. For example, the tilt angle of the escalator at the exit or entrance of the metro station should not be greater than 30°. This type of rule reflects the mandatory requirements that structural components must meet; (2) Existential requirements. For example, if a metro station is not equipped with platform doors, a safety belt should be installed 400 mm away from the edge of the platform. For the second category of rules, the absence of safety belt does not affect the normal use of station facilities. However, installing safety belts can guarantee a safe distance between pedestrians and trains, and enhances the safety of the metro station.

The above DFS knowledge is usually written in natural language, making it difficult to recognize and store by computers. Therefore, to enhance the recognizability of the specification, the specification expressions are split into multiple semantic elements, as shown in Equation (1).

<Semantic elements 1> + <Semantic elements 2> + … + <Semantic elements n> (1)

After the semantic split, the semantic elements are conveniently stored in the DFS database. Semantic labelling is the process of assigning semantic tags to semantic elements in a sentence [41]. Each semantic element is a word or phrase with a semantic label. To structure the specification with semantic labels, this paper defines several semantic labels in the following ways.

**Figure 3:**
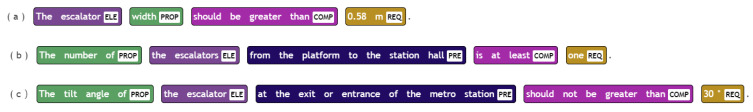
Example of semantic labelling of metro design specification. (**a**–**c**) represents three rules respectively.

Three quantitative requirement rule is shown in Figure 3, and (a), (b) and (c) represents three rules respectively. It consists of five main parts: Properties of Design Elements (PROP), Design Element (ELE), Pre-condition (PRE), Comparative Relation (COMP), Requirement of the Property (REQ). The constraints often contain words, such as “should”, “must”, “should not”, “ strictly prohibit”, etc. They should be converted to the corresponding constraints when constructing a structured design. For example, “should be greater than” can be interpreted as “greater than (>)”. “At least” can be interpreted as “not less than (≥)”. Similarly, “should not be greater than” can be interpreted as “not greater than (≤)”.

Five semantic labels are proposed in this study to represent the different semantics and relationships of words and phrases in the design specifications. The aggregated results are shown in Table 1. “ELE” is used to indicate the element to be designed. Both “PRE” and “PROP” are used to describe “ELE”. The difference is that “PRE” is used to show where the “PRE” is located. The “PROP” is the property to be considered in the design of the “ELE”, such as length and width. The objects referred to by “ELE” under different “PRE” constraints are discrepant. For example, the “ELE” in “escalator at exit or entrance” and “escalator from platform to concourse” are different. In addition, “PRE” may not exist in some simple specifications. The “COMP” is used to describe the comparative or existential relation between the “PROP” and the “REQ”. The “REQ” is the characteristic value that “COMP” corresponding to “ELE” must satisfy.

The semantic labels in Table 1 will be used to build the table named “The specification for the metro station table” in the DFS-KB. In addition, it is the source of DFS specifications for BIM model safety checking.

### 4.2. Identifying DFS Knowledge Elements

**Figure 4:**
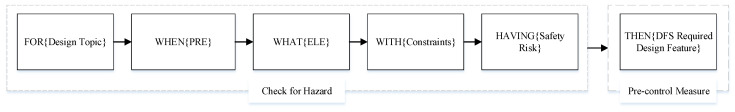
Structure of DFS knowledge logical clause.

By modifying the five-level taxonomy of Nguyen et al. [42], this paper proposes constructing DFS knowledge logic clauses by six elements: Design Topic, ELE, PRE, Constraints, Safety Risks, and Pre-control Measures. The first layer is the design topic, such as metro station engineering, interval engineering, etc. The second layer is the PRE. The third layer is the ELE. The fourth layer is the constraint information related to the design object, which is usually the constraint condition for the risk of occurrence of parameters, such as length, width, angle, etc. The fifth layer is the potential pitfall that can occur under the constraints. The sixth layer is the design measure that should be taken to mitigate the risk, as shown in Figure 4.

The DFS knowledge framework consists of two main parts: The check for hazards and the pre-control measures. The first part focuses on verifying whether the ELE meet the design criteria. The second part focuses on the pre-control measures to be considered for the risks identified in the first part. In the DFS-KB, the first parts are used to build the metro station’s safety risk table. Subsequently, the pre-control measures will be obtained according to the constraints of risk occurrence. The design will be adjusted according to the DFS rules to reduce risks during construction.

The safety-related knowledge in design specifications is usually in the form of constraint-type rules. For example, escalators at the entrances or exits of metro stations may pose a risk if the tilt angle is greater than 30°. Usually, designers design escalators with a tilt angle of 30° or less. Therefore, when designing escalators at station entrances or exits, the following constrain can be set:

Constrain: The tilt angle of an escalator > 30°.

It is easy to be nervous and dizzy when passengers ride escalators with large lifts. The slightest abnormality can easily cause uncomfortable feeling. In addition, elevators with large lifts often require staff to be on-site, which increases costs. If the tilt angle of escalators at the entrances or exits of metro stations is within the above range, a risk event may occur at the escalator. The design measure to address this risk is to control the escalator inclination angle within 30° at design time.

The Section 9.3.5 of the *Code for Design of Metro* stipulates that a safety belt is required at a distance of 400 mm from the edge of the platform when there is no platform door. The width of the safety belt should not be less than 80 mm. The premise that this specification is effective is that the metro in the design does not set the platform door, and the distance from the edge of the platform is less than 400 mm. The platform door may present a “tumble” or “ falling” risk at this time. If the designer and safety manager ignore these constraints, the edge of the station may pose a safety hazard. It can also be translated into constraints for the occurrence of risk events.

Constrain 1: Metro station design without platform doors.

Constrain 2: Distance to the edge of the platform < 400 mm.

If a metro station is designed to meet these constraints, it may pose a “falling” risk. However, different ELE pose different risks under discrepant constraints. A structured framework designed for SK is essential for the query and storage of DFS knowledge. According to the structure of the DFS knowledge framework, the risk identification and pre-control of escalators at station entrances and exits can be translated into the following rule expressions:

FOR{metro station}—WHEN{the exit or entrance of the metro station}—WHAT{escalator}—WITH{The tilt angle of an escalator > 30°}—HAVING{vertigo; falling; uncomfortable}—THEN{The tilt angle of an escalator ≤ 30°}.

Similarly, the DFS knowledge in Section 9.3.5 of the *Code for Design of Metro* can be translated into the following structure:

FOR{metro station}—WHAT{the platform of metro}—WITH{metro design without platform doors, distance to the edge of the platform < 400 mm}—HAVING{tumble; falling}—THEN{400 mm from the edge of the platform should be set for the safety protection belt, and the width of the safety belt in the safety belt should not be less than 80 mm}.

It can be considered that the risk of “tumble” and “falling” may occur in the station project when the metro platform has no door, and the distance from the edge of the platform is less than 400 mm. In the design, a safety belt should be set at 400 mm from the edge of the platform, and a safety belt with a width of not less than 80 mm should be set in the safety belt.

If the ELE meets the first part of the DFS rule in Figure 4, the risk of the ELE has been identified. Then, it is necessary to check whether the ELE complies with the design safety requirements. If the design does not comply with the relevant provisions, it is transferred to the second part of the query pre-control measures to improve the design. If the ELE does not meet the design characteristics and there are no pre-control measures, it must be handled manually by the relevant personnel. Whether the criteria of design features are met or not, they should be registered for subsequent documentation as a process for risk control. In addition, the unrecorded pre-control measures can draw the attention of relevant personnel to improve the DFS-KB continuously.

### 4.3. Building DFS-KB

**Figure 5:**
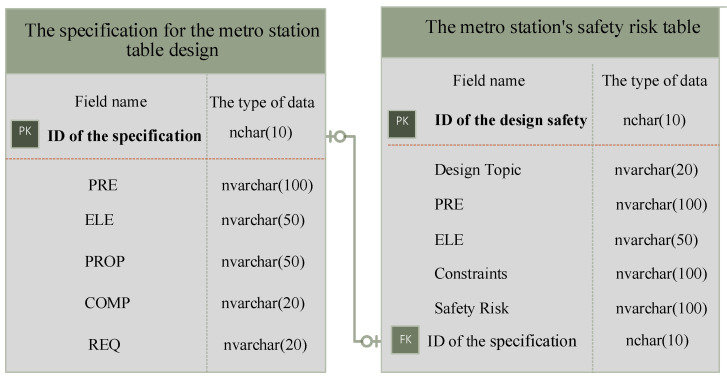
The KB stores tables and relationships between tables.

The identified design SK is stored in the DFS-KB in the form of tables. In this paper, two types of tables are established to store SK: The specification for the metro station table design and the metro station’s safety risk table. The specification for the metro station table design is split into several semantic elements using the semantic labels in Section 4.1. These semantic elements are used to build the table, which is named “The specification for the metro station table design”. The metro station’s safety risk table is created based on the DFS knowledge elements identified in Section 4.2. The metro station’s safety risk table presents the secure design information that needs to be satisfied by the ELE under different constraints. In addition, the identified design safety risks are stored in the metro station’s safety risk table. The constituent elements of each data table are shown in Figure 5.

The DFS knowledge in the different tables is linked by defining the correspondence between data tables. PRIMARY KEY (PK) and FOREIGN KEY (FK) are used to ensure the integrity of the database and to link different data tables. The presence of PK and FK makes different data tables accessible to each other. The PK is unique in the table and is used as an identifier for each row of data. The FK reflects the binding effect of another data table on this data table. The FK is usually the PK of another table. The value of the FK is derived from the PK of the table to which it is linked.

For example, in the specification for the metro station table design, the ID of the specification is set to the PK. Then, the ID of the specification is inserted into the metro station’s safety risk table. At this point, the PK becomes a FK in the metro station’s safety risk table. A correspondence is established between the specification for the metro station table design and the metro station’s safety risk table. In addition, the data in the specification for the metro station table design can be accessed in the metro station’s safety risk table.

### 4.4. The Query of DFS Knowledge

**Figure 6:**
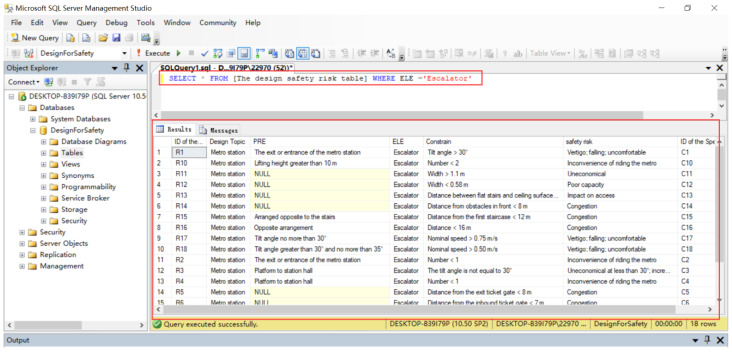
Example of KB query.

SQL will be used to implement the query of SK (Yuan et al., 2019). SQL is easy to master, and designers and safety managers can master the retrieval of DFS knowledge with only simple training. Figure 6 shows an example of retrieving escalator DFS information using SQL statements.

## 5. Application of DFS-KB Integrated with the Revit Platform

The metro station design work is completed based on national, departmental, and industry standards. The design SI is one of the extremely important stages of metro station design. In addition, it aims to check whether there are any safety problems with the design. Moreover, it can reduce the safety risk in the construction phase. The process of safety rule checking can be roughly divided into four stages:

(1) Rule interpretation. Security regulations, usually in text form, need to be translated into computer-recognizable rules, and the rules need to be linked to ELE.

(2) Check the design property requirements of the object. Reliable automated design safety checks require the provision of design information in the model. This information includes the design object’s name, attributes, and location.

(3) Execution of checking. Rule execution connects the rule base with the building information model. By calling the requirements of component design attributes in the rule base, the information of building component attributes in the BIM model is judged. In addition, the building objects that do not meet the design requirements are screened out.

(4) Output the checking results [37]. The output of the inspection results is reported in two different forms: (a) A visual alerting window showing information about the inspected risk components and giving precautions to mitigate these risks; (b) Visualization of the location of the risk components. With form (b), the designer and safety managers can quickly find the location of the risk components and improve the design based on the precautions given in (a).

### 5.1. Data Preparation

The essential data required for design checking consist of two main types: (1) DFS specification; (2) ELE attribute data.

The SK in the design specification needs to be obtained before design checking and transformed into rules that can be recognized by the computer, as described in Section 4.1. In addition, the design specifications need to be transformed into a logical structure to be queried by the automatic drawing inspection system. The invoked rules are used as guidelines for checking the design. In this paper, the design specifications are organized in logical clauses, and the DFS knowledge is stored in the KB. This part is described in Section 4.2.

Moreover, the attribute of ELE is the essential preparation for SI. The BIM model is the information carrier for engineering features, metro design, and regulations. The core perspective of BIM technology is to use digitalization and information technology to provide a building information base that is consistent with the actual situation of the project. The model contains information, such as properties of structural components, the interaction between components and the surrounding environment, and graphical parameters, which provides ELE property data for design checking.

### 5.2. The Process of Inference and Checking

The reasoning and inspecting plug-in consists of three main parts: (1) Querying ELE property information from Revit; (2) Querying rule information from the SQL Server database; and (3) Executing checking and outputting results. Microsoft Visual Studio (VS) is an application development environment developed by Microsoft Corporation. In this paper, VS is used to extract ELE instance information and judgment on whether they are in accordance with design specifications.

The graphical and parametric data of the ELE required for the inspection plug-in are extracted from the model through the Revit API. The DFS rules in the KB are queried in Visual Studio using SQL. Therefore, it is necessary to reference Revit API.dll and Revit APIUI.dll in the development program to invoke the methods and functions in the Revit API. In addition, the reference code should be written in the namespace. Attributes must be referenced in the namespace when querying the ELE attribute information in Revit. The main namespace reference code is as follows:
**Algorithm 1** Namespace1. using Autodesk.Revit.Attributes;  // For querying ELE property information 2. using Autodesk.Revit.DB;     // For querying ELE property information 3. using Autodesk.Revit.UI;     // For querying ELE property information 4. using System.Data.SqlClient;   // Used to retrieve database information

(1) Retrieve ELE property data from Revit

The extraction of attribute information in Revit usually requires sifting through all components in the model. ELE that meets the screening criteria is filtered out and stored in a collection. This process is usually carried out by creating a collector in Visual Studio and using FilterElementcollector to filter out the ELE to be checked. Revit LookUp is a common property viewing tool used when developing Revit plug-ins. The property information required for design inspection can be called from Revit LookUp using the LookUpParameter function. The following algorithm illustrates the process of using the above method to filter the ELE named “Entrance or Exit Escalator 2” from Revit and call its design angle parameter.
**Algorithm 2** Retrieve ELE property data1. FilteredElementCollector collector = new FilteredElementCollector(doc);  // Creating a collector 2. FamilyInstance escalator =new 3. FilteredElementCollector(doc).OfCategory(BuiltInCategory.OST_GenericModel).OfClass(typeof(FamilyInstance)). 4. FirstOrDefault(x => x.Name == “ Entrance or Exit Escalator 2”) as FamilyInstance;  // Filter out family-Instance 5. string escalatorAngel = escalator.Symbol.LookupParameter(“tilt angle”).AsValueString();  // Extraction of family 6. instance parameters

(2) Retrieve SQL database specification information

Safety specifications in the database can be accessed through SQL statements. Since the VS platform has good compatibility with different languages, the SQL statements in the VS platform can be carried out to query the rule data. Database connection statements need to be written before calling the database. The connection statement needs to indicate the server domain name, database name, user name, and user password of the database. The database connection algorithm is as follows.
**Algorithm 3** Retrieve SQL database1. string connString = @”Server=.;DataBase=DesignForSafety;Uid=sa;Pwd=lvtao6686”;  // Basic 2. information of the database 3. string sql = $”SELECT Attribute FROM DFSrules WHERE ELE LIKE ‘% Escalator %’”;  // Statements for 4. querying DFS rule information 5. SqlConnection conn = new SqlConnection(connString);    //Establishing database connection 6. conn.Open();                     // Open connection 7. …                          // DFS rule query statement 8. conn.Close();                     // close connection

(3) Implementation of rule checking

**Figure 7:**
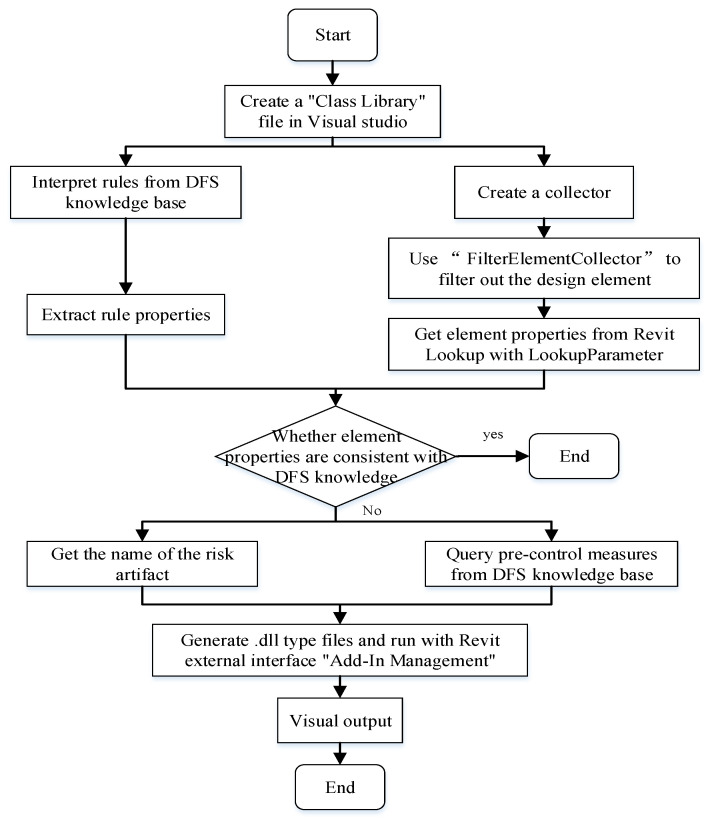
The development process of the inspection plug-in.

Figure 7 describes the development process of the inspection plug-in. The inspection process determines whether the ELE properties are consistent with the DFS knowledge. The judgment process is performed by adding the constraint information of the component’s properties to the “if (constrain)” statement. If the constraint information is judged to be true, the component may generate the safety risk. At this time, it is necessary to retrieve the pre-control measures to deal with the design risk from the DFS-KB. In addition, the screened risk component IDs are added to the highlighted set for the visual output of risk inspection results.

As an example, consider a metro station escalator. The safety design requirement is to control the escalator angle within 30°, and the judgment condition in the if statement can be set to “angle > 30”. If the result of “angle > 30” is “true”, the escalator is judged to be a risky component. The precautions to improve the design need to be retrieved from the KB, and highlight the model’s risk components. After the algorithm is completed, it is compiled and run in VS to generate a .dll file that can be used in Revit.

### 5.3. Output the Inspection Results

Before performing the BIM model design SI, the .dll file needs to be loaded manually through the External Command “Add-In Manager” in Revit. This process only needs to be added once manually to start the automatic inspection. The result of the inspection is displayed in Revit as an alerting window, including the name of the risk component, the safety risk, and the pre-control measure to improve the design. The risk components are marked in the BIM model by distinctive colors. For the inspected risks, the designer can modify the attribute information of the ELE according to the design suggestions provided by the system. Then, safety risks in the construction are eliminated or avoided in the design phase.

## 6. Case Study

### 6.1. Project Overview

Escalators in metro stations are a vital tool for the transfer of passengers, and they are the location where metro accidents often occur. The validity of the KB and inspection plug-in is verified in a metro station in Lanzhou. The station type is an underground two-story island platform. The station is located in the center of the city, surrounded by large shopping malls with high pedestrian flow. The station contains six escalators, including three escalators at the entrances and three escalators connecting the station platform to the concourse. The escalators are responsible for carrying the main passengers of the station. Safety is essential before the construction process. Therefore, the specifications for the design of metro, journal literature, and expert knowledge related to the escalators are used to establish a DFS-KB using the structured approach introduced in this paper. In addition, the DFS-KB is combined with BIM technology to identify escalator safety hazards.

### 6.2. Building the DFS-KB

Most of the specifications for escalators in metro stations are attribute constraints. These constraint specifications guarantee the safety of the stations. Metro design safety standards and construction safety management codes are the summation and condensation of many years of experience in metro safety management; therefore, combining codes is an effective way to obtain safety standards [43]. This study focuses on the principal codes promulgated for the design and construction safety management of metro projects in China, as shown in Table 2. Then, literature search and expert research are combined to sort out the rules related to the safety of escalators in metro stations. A specification for the metro station table design was created according to the method described in Section 4.1 to store the metro design specifications processed by semantic annotation, as shown in Table 3.

For the safety rules associated with escalators identified in Table 3, the limiting conditions that may cause the risk are determined using the method described in Section 4.2. Expert experience is combined to identify the risks that may occur for the design under the limiting conditions, as shown in Table 4.

Tables are linked by PK and FK. In addition, the data can be queried directly from each other. The specification for the metro station table design and the metro station’s safety risk table constitute the DFS-KB. The established DFS-KB will be used as design guidelines for the design of SI.

### 6.3. Application of SI Plug-In

**Figure 8:**
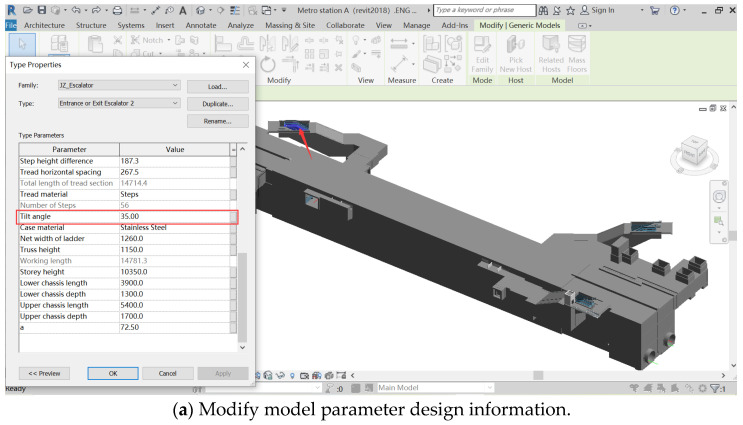

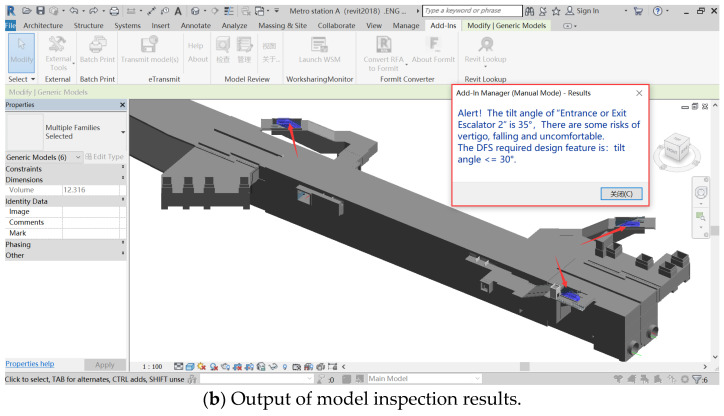
Demonstration of the effects of automated inspection.

To verify the effectiveness of the design SI plug-in, some parameters in the BIM model are specially set. In this case, the “tilt angle” property of the element named “Entrance or Exit Escalator 2” is set to 35°, as shown in Figure 8a. The rest of the design properties are not specially handled. As a result, escalators with an angle greater than 30° were identified and selected after the plug-in was run. It is shown in bright blue, which corresponds to the position indicated by the red arrow in Figure 8b. In this case, the escalator tilt angle is quite large (greater than 30°), and there is a risk of “vertigo”, “falling”, and “uncomfortable” during use. In addition, the DFS rule logic sentences should be in the following format:

FOR{metro station}—WHEN{the exit or entrance of the metro station}—WHAT{escalator}—WITH{The tilt angle of an escalator > 30°}—HAVING{vertigo; falling; uncomfortable}—THEN{The tilt angle of an escalator ≤ 30°}.

The mentioned inspection process is completed automatically in the SI plug-in. The constraints in “WITH” are judged with an if statement during the checking process. If the result is “true”, then the ELE is at risk of vertigo, falling, and uncomfortable during use. Therefore, in order to reduce this risk, the measures to be taken are usually to control the tilt angle of the escalator to 30° or less. In this case, the plug-in outputs the pre-control measures to mitigate the risk in an alerting window, as shown in Figure 8b. The designer can improve the design in the property operation panel based on the pre-control measures to reduce the risk during construction.

## 7. Discussion

Studies have shown that DFS can prevent many accidents [3,10]. Therefore, reducing metro safety accidents by design provides ideas for metro safety management [15]. A wealth of knowledge related to DFS are embedded in metro design specifications and journal literature. In addition, the experience gained by experienced engineers is crucial to improving the design. However, most of this knowledge is scattered in various texts and recorded in natural language, making it difficult to share and reuse. In addition, it is not easy to integrate SK with information technology.

Some scholars have focused their research on translating safety specifications into a form that computers can store and use. For example, Zhou et al. [41] used natural language processing methods to represent concepts and relationships in specification texts. Malsane et al. [20] transformed human-readable residential fire specifications into computer-executable rules. Zhang and El-Gohary [18] used semantic and machine learning approaches to extract building specifications from textual specifications. These studies mainly focus on the extraction and storage of specifications. However, studies on the informative use of the rules are lacking.

Regarding risk automation inspection, some studies tabulated the feasibility of using KB and BIM technology to solve safety design problems. Lu et al. [45] used SK from construction specifications to achieve SI. Yuan et al. [16] used KB and BIM technology to automate inspection safety risks in construction. However, most of these studies focus on falling accidents in the construction industry. In addition, few scholars have studied the safety issues in the design process of metro stations.

Therefore, this paper proposes a structured method for identifying SK. The method is used to establish a safety KB for the design process of metro stations. This paper realizes the information-based storage, transmission, and use of SK. At the same time, the KB is combined with BIM technology to realize the automated inspection and risk visualization output of metro station design. It assists designers and safety managers in improving the design and reduces the safety design problems caused by their insufficient knowledge reserve and negligence, etc. At the same time, this study improves the visualization and convenience of metro safety design and saves labor costs for the design work. A comparison of the ground body DFS-KB proposed in this paper with previous studies is shown in Table 5.

However, the established DFS-KB also has some limitations. First, not all metro design specifications and journal literature can be stored using the proposed security rule identification method. Some safety rules need to be combined with expert experience to refine the safety rules. Second, when storing safety regulations in the DFS-KB, the safety regulations in the text need to be analyzed and converted into a computer-recognizable table form. This process needs to be carried out manually. However, this work only needs to be executed once when building the KB. The KB can be improved similarly when the safety regulations change. If the DFS knowledge is not included in the KB, the automated checking system of the integrated BIM software cannot perform automatic design inspections. In this case, it needs to be handled manually by designers. Through the continuous improvement and supplementation of the DFS-KB, these problems will be solved and applied to future designs.

## 8. Conclusions

Designers and safety managers are critical to reducing risk during construction. However, there is a lack of appropriate SK sharing and reuse methods to compensate for the designer’s imperfect KB and lack of design experience, which is a considerable hindrance to improving the design quality. Therefore, this paper proposes a structured SK identification and storage method to transform national specifications and journal literature in natural language form into structured SK. A DFS-KB is established with the extracted security knowledge to realize the sharing and reuse of security knowledge. In addition, the database is extended to BIM software to help designers automatically check the safety risks in the design, and to improve design quality with knowledge-based decision-making, allowing designers and safety managers to focus on problem-solving rather than problem identification. Moreover, the inspection results are presented to designers and safety managers in a visualized form, enhancing the visualization of risks in the design process and reducing the labor cost for the design phase. The KB query function is shown in Figure 6. The design inspection results of the KB applied to engineering practice are shown in Figure 8. As seen from the illustrative case, when designers use the SI plug-in in the 3D model, ELE with hidden risks are marked and highlighted in the 3D model, enabling risk visualization. The designer can take the necessary design measures to mitigate the risks based on the prompted pre-control measures during the design phase.

Although this study has shortcomings, a structured method for storing and reusing security knowledge is provided. The method is used to build a DFS-KB for the design process from SK in the metro design specifications, journal literature, and expert experience. The DFS-KB provides a way for designers and safety managers to store and query DFS knowledge. It has been well applied in the metro design and inspection work. As the KB continues to improve, the accuracy of the risk automation checking system that relies on the DFS-KB will significantly improve. Future work will continue to improve the study in three ways: (1) Enriching the sources of the DFS-KB; (2) Automating the extraction of security knowledge from national specifications and journal literature; (3) Incorporating other types of DFS knowledge into the KB; and (4) Automating risk inspection of complex rules.

## Figures and Tables

**Table 1:** Semantic labels.

Name	Definition	Examples
PRE	Used for modifying and positioning “ELE”. It is not necessary.	Station entrances or exits, platform-less doors, etc.
ELE	Object to be designed/inspected.	Escalators, safety belts, station platforms, etc.
PROP	The “ELE” property to be designed.	Existence, angle, length, etc.
COMP	To describe the comparison/existence relationship between “PROP” and “REQ”.	Greater than, equal, no more than, at least, etc.
REQ	Characteristic values that “COMP” must meet.	30°, 400 mm, 5 m, etc.

**Table 2:** Principal codes for the design of metro.

Code	Abbreviation
Code for Design of Metro	GB 50157
Technical code of urban rail transit	GB 50490
Technical code for fire safety of construction site	GB 50720

**Table 3:** The specifications for the metro station table design.

ID of the Specification	PRE	ELE	PROP	COMP	REQ	Source
C1	The exit or entrance of the metro station	Escalator	Tilt angle	≤	30°	GB 50157, GB 50490
C2	The exit or entrance of the metro station	Escalator	Number	≥	1	GB 50157, Investigation
C3	Platform to station hall	Escalator	Tilt angle	=	30°	GB 50157, GB 50720
C4	Platform to station hall	Escalator	Number	≥	1	GB 50157, Investigation
C5		Escalator	Distance from the exit ticket gate	≥	8 m	GB 50157, GB 50490
C6		Escalator	Distance from the inbound ticket gate	≥	7 m	GB 50157, GB 50490
C7	The width is 1 m	Escalator	Capacity	≤	8190 people per hour	GB 50157, GB50720
C8	The width is 0.65 m	Escalator	Capacity	≤	5265 people per hour	GB 50157, GB50720
C9	Lifting height greater than 6 m	Escalator	Number	≥	1	Investigation [12]
C10	Lifting height greater than 10 m	Escalator	Number	≥	2	Investigation [12]
C11		Escalator	Width	≤	1.1 m	GB 50157 [15]
C12		Escalator	Width	≥	0.58 m	GB 50157 [15]
C13		Escalator	Distance between flat stairs and ceiling surface	≥	2.3 m	GB 50720
C14		Escalator	Distance from frontal obstacles	≥	8 m	GB 50720
C15	Arranged opposite the stairs	Escalator	Distance from the first staircase	≥	12 m	GB 50157
C16	Opposite arrangement	Escalator	Distance	≥	16 m	GB 50157
C17	Tilt angle no more than 30°	Escalator	Nominal speed	≤	0.75 m/s	GB 50157 [15]
C18	Tilt angle greater than 30° and no more than 35°	Escalator	Nominal speed	≤	0.50 m/s	GB 50157 [15]
C19	Escalators with a speed of 0.5 m/s	Flat stairs	Number	=	3	GB 50157, Investigation [44]
C20	Escalators with a speed of 0.65 m/s	Flat stairs	Number	≥	3	GB 50157, Investigation [44]
C21	Escalators with a speed of 0.75 m/s	Flat stairs	Number	≥	4	GB 50157, Investigation [44]
C22	Escalator	Stairs	Width	≥	0.38 m	GB 50157
C23	Escalator	Stairs	Height	≥	0.24 m	GB 50157
C24	Escalator	Handrail	Height	≥	0.9 m	GB 50157
C25	Escalator	Handrail	Height	≤	1.1 m	GB 50157
C26	Escalator	Handrail	The horizontal distance between the outer edge and decorative surface	≥	80 mm	Investigation
C27	Adjacent escalators	Handrail	The horizontal distance between outer edges	≥	160 mm	Investigation

**Table 4:** The metro station’s safety risk table.

ID of the Safety Risk	Design Topic	PRE	ELE	Constrain	Safety Risk	ID of the Specification
R1	Metro station	The exit or entrance of the metro station	Escalator	Tilt angle > 30°	Vertigo; Falling; Uncomfortable	C1
R2	Metro station	The exit or entrance of the metro station	Escalator	Number < 1	Inconvenience of riding the metro	C2
R3	Metro station	Platform to station hall	Escalator	The tilt angle is not equal to 30°	Uneconomical at less than 30°; Increased risk of falling at greater than 30°	C3
R4	Metro station	Platform to station hall	Escalator	Number < 1	Inconvenience of riding the metro	C4
R5	Metro station		Escalator	Distance from the exit ticket gate < 8 m	Congestion	C5
R6	Metro station		Escalator	Distance from the inbound ticket gate < 7 m	Congestion	C6
R7	Metro station	Width is 1 m	Escalator	Capacity > 8190 people per hour	Exceeding escalator capacity	C7
R8	Metro station	Width is 0.65 m	Escalator	Capacity > people per hour	Exceeding escalator capacity	C8
R9	Metro station	Lifting height greater than 6 m	Escalator	Number < 1	Inconvenience of riding the metro	C9
R10	Metro station	Lifting height greater than 10 m	Escalator	Number < 2	Inconvenience of riding the metro	C10
R11	Metro station		Escalator	Width > 1.1 m	Uneconomical	C11
R12	Metro station		Escalator	Width < 0.58 m	Poor capacity	C12
R13	Metro station		Escalator	Distance between flat stairs and ceiling surface < 2.3 m	Impact on access	C13
R14	Metro station		Escalator	Distance from frontal obstacles < 8 m	Congestion	C14
R15	Metro station	Arranged opposite to the stairs	Escalator	Distance from the first staircase < 12 m	Congestion	C15
R16	Metro station	Opposite arrangement	Escalator	Distance < 16 m	Congestion	C16
R17	Metro station	Tilt angle no more than 30°	Escalator	Nominal speed > 0.75 m/s	Vertigo; Falling; Uncomfortable	C17
R18	Metro station	Tilt angle greater than 30° and no more than 35°	Escalator	Nominal speed > 0.50 m/s	Vertigo; Falling; Uncomfortable	C18
R19	Metro station	Escalators with a speed of 0.5 m/s	Flat stairs	Number not equal to 3	Shorter escalator change time; Falling	C19
R20	Metro station	Escalators with a speed of 0.65 m/s	Flat stairs	Number < 3	Shorter escalator change time; Falling	C20
R21	Metro station	Escalators with a speed of 0.75 m/s	Flat stairs	Number < 4	Shorter escalator change time; Falling	C21
R22	Metro station	Escalator	Stairs	Width < 0.38 m	Less space for people to stand; Falling	C22
R23	Metro station	Escalator	Stairs	Height < 0.24 m	Uneconomical	C23
R24	Metro station	Escalator	Handrail	Height < 0.9 m	Inconvenient gripping; Falling	C24
R25	Metro station	Escalator	Handrail	Height > 1.1 m	Inconvenient gripping; Uneconomical	C25
R26	Metro station	Escalator	Handrail	The horizontal distance between the outer edge and decorative surface < 80 mm	Hands will rub against the decorative surface when holding the handrail	C26
R27	Metro station	Adjacent escalators	Handrail	The horizontal distance between outer edges < 160 mm	Mutual influence when holding the handrails	C27

**Table 5:** Comparison in this study and previous researches.

Research of DFS	Field of Study	Semantic Labels	Natural Language Processing	Knowledge Base	Inspection Plug-In for BIM
Malsane et al. [20]	Building Firefighting		√		
Zhang et al. [24]	Building Construction	√	√		
Lu et al. [45]	Building Construction				
Yuan et al. [16]	Building Construction			√	√
This paper	Metro station design	√	√	√	√
